# The Experience of Deconversion Among Polish Catholic Adolescents: A Mixed-Methods Investigation

**DOI:** 10.1007/s10943-025-02428-x

**Published:** 2025-08-29

**Authors:** Michał Grupa, Beata Zarzycka

**Affiliations:** Institute of Psychology, The John Paul II Catholic University of Lublin, Al. Racławickie 14, 20-950 Lublin, Poland

**Keywords:** Deconversion, Religion, Anxiety, Adolescents, Catholicism, Mixed-methods study, Longitudinal design

## Abstract

This study aimed to address a gap in the literature concerning the emotional dynamics of deconversion among adolescents, specifically focusing on the relationship between deconversion, anxiety, and the prior centrality of religion. Using a mixed-methods approach, the study combined qualitative research to uncover the lived experiences of Polish Catholic adolescents who have undergone deconversion, with a longitudinal quantitative design to examine changes in anxiety over time in relation to the initial centrality of religion. In Study 1, five adolescents (aged 15–18; *M* = 16.4*, SD* = 1.14) participated in in-depth, semi-structured interviews, which were analyzed using thematic analysis. Three main themes were generated from the data: Confronting Turmoil at the Beginning, Mourning the Loss of Foundational Beliefs, and Progressing toward Adaptation. In Study 2, 268 adolescents (aged 13–16 at Time 1) participated in a longitudinal quantitative study consisting of three measurement points, which utilized a linear mixed model (LMM) to investigate the relationships between anxiety, deconversion, time, and the centrality of religion. The results indicated that the link between deconversion and anxiety was strongest initially and decreased over time, particularly for adolescents who had previously placed high importance on religion. The findings highlight the emotional dynamics of adolescent deconversion and suggest that the prior centrality of religion plays a significant role in shaping the emotional impact of this process.

**Supplementary Information:**

The online version contains supplementary material available at 10.1007/s10943-025-02428-x.

## Introduction

Contemporary sociocultural transformations, along with an increasing trend toward secularism, indicate that the influence of religion on individuals and communities is diminishing. In Europe, where religion has historically played a significant role in shaping cultural identity, there is a noticeable decline in participation in religious practices, alongside a reduced emphasis on religion in everyday life (Ipsos, 2023). Similar patterns are evident in Poland, where younger generations are increasingly distancing themselves from faith, traditions, and the practice of religious rituals, while still maintaining a formal affiliation with the Catholic Church (CBOS, 2024).

Adolescents represent the most vulnerable demographic to deconversion, defined as the process of departing from faith. This developmental stage is characterized by an intense quest for identity, a reevaluation of personal values, and a rigorous examination of societal and familial expectations (Magaldi-Dopman & Park-Taylor, 2010). During this period, individuals often grapple with the dissonance between their beliefs and external pressures, critically reassessing the relevance of religion and its influence on their convictions and lives (King et al., 2013).

For many individuals, deconversion is a complex and emotionally charged experience, often involving feelings of relief along with challenging emotions such as anxiety, guilt, and internal conflict (Grupa & Zarzycka, 2025; Jindra & Lee, 2023; Lee & Gubi, 2019). This process can be particularly difficult in societies where religion is a significant cultural norm. Young individuals who move away from religious affiliations frequently encounter family rejection, significant alienation, or difficulties in building a new support network (Nica, 2019). These challenges bring up questions regarding the relationship between deconversion and anxiety. Is deconversion related to anxiety? How do the processes of deconversion relate to anxiety over time? What impact does previous religiousness have on this process?

To address these questions, the present study examined the psychological dynamics of youth deconversion using a mixed-methods design. In the first phase, qualitative interviews were conducted and thematically analyzed to construct key experiential themes associated with deconversion. These qualitative insights then informed the structure of the second, longitudinal quantitative phase, which assessed anxiety levels and prior religiousness across three time points, allowing us to explore how anxiety changes over time in relation to individuals’ earlier degree of religious centrality.

### The Emotional Trajectory of Deconversion

Deconversion is defined as “the change of a person’s religious orientation in a specific biographical time which involves re-writing one’s religious identity, revising one’s system of beliefs and world views, and restructuring one’s way of thinking, moral judgment, and dealing with authority—with a special focus on the act of leaving the old and searching for something different” (Streib et al., 2009, p. 23). Deconversion encompasses (1) the loss of religious experiences such as meaning, purpose, and connection with God; (2) a rise in intellectual skepticism and rejection of previously held beliefs; (3) a critical examination and dismissal of moral values; (4) emotional distress that includes grief, guilt, despair, and loneliness; and (5) separation from faith communities and their practices (Streib & Keller, 2004). Thus, deconversion is a complex process encompassing emotional, intellectual, and moral aspects, representing a dynamic change beyond mere shifts in beliefs and disconnection (Paloutzian et al., 2013).

The emotional aspects linked to the deconversion process display significant complexity and variability. Individuals undergoing deconversion frequently experience a profound sense of relief as they liberate themselves from the doctrines and beliefs that previously constrained them. Qualitative research indicates that participants aimed to investigate viewpoints and possibilities outside their religious affiliations (Fazzino, 2014; Lee & Gubi, 2019; Nica, 2020). This tendency, which led to deconversion, brought about a deep sense of freedom. However, the experience of liberation also has a dark side. Findings from qualitative (Adam, 2009; Grupa & Zarzycka, 2025; Jindra & Lee, 2023; Nica, 2020) and quantitative studies (Fenelon & Danielsen, 2016) suggest that individuals who have undergone deconversion experience fears of stigma and diminished social support. They also confront challenges to their established self-perceptions and psychological distress. Moreover, qualitative research (Adam, 2009; Lee & Gubi, 2019) indicates that individuals who distanced themselves from religious affiliations experienced particular emotional responses, such as anxiety, guilt, and fear of social rejection related to deconversion.

The emotional trajectories related to deconversion may evolve. Initially, the emotional intensity associated with the deconversion process is considerably heightened (Adam, 2009; Davidman & Greil, 2007), but its intensity diminishes over time (Jindra & Lee, 2023; Nica, 2020). Individuals adjust to a new lifestyle without a religious framework. A longitudinal study by Streib and Keller (2009), based on interviews, indicated that people experiencing deconversion gradually develop a greater sense of purpose in life and increased self-acceptance. Adopting a non-religious identity can help alleviate negative feelings (Nica, 2020). This process varies significantly among individuals, resulting in diverse outcomes. Some individuals may encounter persistent difficulties, such as ongoing feelings of guilt or tense family dynamics (Nica, 2019). Therefore, the emotions associated with deconversion progress from deep pain and uncertainty at first to adjustment and gradual healing. Time is essential for managing this intricate and multifaceted process.

### Navigating Deconversion in Religious Societies

The emotional trajectories associated with deconversion can be greatly intensified in settings where religion has a significant influence. This is evident in countries such as Poland, where a large portion of the population adheres to religious beliefs, making faith an integral aspect of the dominant culture. Christianity, and more specifically, Catholicism, exerts a profound influence on the cultural and national identity of Poland. The Roman Catholic Church serves as a pivotal institution within both the religious and social spheres, impacting various dimensions of social life (Mariański, 2019). Poland demonstrates significant homogeneity in religious affiliation (Żemojtel-Piotrowska et al., 2021), with around 71% of the population identifying as Roman Catholic (CBOS, 2021). The dominance of a singular religion within a sociocultural framework can substantially influence deconversion processes. Deconversion goes beyond being merely a personal journey; it also involves social challenges as individuals transition from a majority to a minority group. Given the considerable religious uniformity in society, individuals face limited options, making their deconversion and adjustment to a new social environment more complicated.

Deconversion among young individuals frequently leads to significant value conflicts. Those who withdraw from their faith face pressure from family and society, revealing a clash between established traditions and contemporary beliefs (Mariański, 2021). Deconversion involves seeking autonomy in forming one’s perspective. This process usually results in increased distance from the Church and a rejection of moral standards grounded in religious doctrine. Abandoning faith often leads to losing their religious community and feeling detached from family customs and collective rituals. This departure can create tension with family members who still adhere to the faith, intensifying feelings of isolation within the family unit (Fazzino, 2014). Quantitative research demonstrates a correlation between deconversion and insufficient familial support (Łysiak et al., 2020). Qualitative research includes narratives that often express feelings of alienation, guilt, and social isolation associated with abandoning one’s faith (for a review, see Hardy & Taylor, 2024). Emotions are dynamic and may evolve as people adjust to new identities and relationships (Lee & Gubi, 2019; Streib, 2021).

### Adolescence: A Transformative Stage for Exploring Faith

Adolescence is a distinct stage of human development characterized by biological, psychological, and social changes that create a supportive environment for exploring religious issues. This includes seeking meaning and purpose in life, making lifestyle choices, experiencing transcendence, and questioning previously accepted worldviews and values (King et al., 2013; Paloutzian et al., 2013). During this stage, adolescents evaluate their beliefs and values while considering the cultural messages received from their families (Magaldi-Dopman & Park-Taylor, 2010). They may resist the established order, which can manifest in changes in religious group membership, the adoption of a rationalist perspective on life, or the rejection of religion (Nowosielski & Bartczuk, 2017). Adolescents often experience increased emotional sensitivity, particularly in relation to peer pressure and heightened conflicts with their parents. Cognitive development lags behind biological growth, leaving adolescents vulnerable to emotional tensions from cognitive processes and social interactions (Briggs, 2008). A critical approach to religion and questioning traditional religious structures has become increasingly common among adolescents in Poland (CBOS, 2021; Mariański, 2021). Challenging religious norms and analyzing the moral values from religious teachings promotes unity among adolescents, enhancing their sense of belonging and group identity (Zarzycka et al., 2022).

### The Present Study

Although earlier studies have significantly advanced the understanding of the psychological dimensions of religiousness and its relationship to anxiety (for a review, see Stewart et al., 2019), the role of anxiety in the specific context of deconversion has received comparatively little attention. Existing research tends to focus on related constructs, such as religious doubt or spiritual struggle (Wilt et al., 2016; Zarzycka et al., 2023), often overlooking a direct examination of anxiety as it emerges during the process of leaving one’s faith (e.g., Zarzycka et al., 2023). Yet, deconversion involves not only the cognitive process of deidentification but also a distinct experiential and emotional dynamic, in which anxiety may play a central role (Adam, 2009; Jindra & Lee, 2023; Nica, 2020).

The operationalization of these processes in empirical research presents significant challenges, particularly when using a statistical approach. This difficulty arises from the inherent complexity and subjectivity of deconversion, which often unfolds as a gradual, nonlinear, and emotionally nuanced experience. Standardized measures may fail to capture the personal meanings and internal tensions that characterize such transitions. In contrast, a qualitative approach—especially one focused on examining biographical experiences—offers greater potential for identifying key themes, emotional trajectories, and interpretive frames (Mariański, 2019). These insights can then inform the design of quantitative instruments and guide the formulation of hypotheses, contributing to more context-sensitive and theoretically grounded statistical analyses. However, such qualitative work has so far been applied primarily to non-Catholic contexts (Davidman & Greil, 2007), leaving a gap in understanding deconversion in culturally Catholic populations. Therefore, we structured our mixed-methods investigation to utilize an exploratory sequential design, where qualitative data were collected first to explore the phenomenon and to support subsequent quantitative research (Creswell, 2012).

In the first phase (Study 1), we conducted qualitative interviews to explore participants’ subjective experiences of deconversion. This approach enabled us to uncover rich, context-specific insights and to identify anxiety as a salient emotional theme within the deconversion process. These qualitative findings then informed the design of the second phase (Study 2), a longitudinal survey aimed at examining the relationship between deconversion, anxiety, prior religious salience, and time since disaffiliation (Creswell, 2012; Rendle et al., 2019). By grounding the quantitative component in qualitative data, the study offers a more nuanced and theoretically coherent analysis of the emotional dynamics of youth deconversion.

## Study 1. A Qualitative Study of the Emotional Process of Youth Deconversion

The aim of the qualitative study was to explore how adolescents experience deconversion, with particular attention to the emotional dimension of anxiety.

### Method

#### Participants and Procedure

The qualitative data presented in this article derive from the pilot phase of a broader research project on adolescent deconversion from Roman Catholicism in Poland. The Research Ethics Committee at the authors’ university approved the project (Decision number KEBN 21/2022). During this pilot phase, semi-structured interviews were conducted with five adolescents who were former Catholics, living in various urban and rural areas of Poland. The participants included two males and three females, aged between 15 and 18 years (*M* = 16.4, *SD* = 1.14). Participant characteristics are presented in Table 1.

**Table 1:** Demographic characteristics of participants in study 1

Variable	Participant 1	Participant 2	Participant 3	Participant 4	Participant 5
Gender	Women	Women	Women	Man	Man
Age	15	16	17	16	18
School	High	High	Technical	Technical	Technical
School location	City < 50.000	City < 50.000	City 50.000–200.000	City < 50.000	City 50.000–200.000
Residence	Village	City < 50.000	City 50.000–200.000	City < 50.000	City 50.000–200.000
Religion	Roman Catholic	Roman Catholic	Roman Catholic	Roman Catholic	Roman Catholic
Time from deidentification (months)	12	18	17	10	35

This pilot study was originally designed to refine the interview procedure and explore preliminary themes ahead of the main qualitative phase (Creswell, 2012). However, the interviews yielded unexpectedly rich and thematically dense data, particularly concerning the emotional experience of anxiety. The narratives offered valuable insight into how feelings of anxiety developed and changed over time in the context of leaving religion. Participants also emphasized the personal and social importance of religion, describing it as a central part of their identity and a significant value within their family environments. They were not only open and willing to share their experiences, but also notably articulate in expressing the psychological and existential complexity of their stories.

Recognizing the analytical value of this emergent theme, we decided to pursue it further through in-depth interpretive analysis. This decision was grounded in the open and flexible logic of qualitative research (Patton, 2015) and supported by the principles of purposeful sampling and information power (Malterud et al., 2016). Although the sample was small, it provided sufficient depth, relevance, and specificity to address the interpretive aims of the study (Hennink et al., 2017; Young & Casey, 2019). Moreover, as the participants formed a relatively homogeneous group, thematic saturation could be achieved with fewer interviews (Guest et al., 2020).

The interview, consisting of seven open-ended questions, was designed to explore adolescents’ experiences of deconversion. Interviews lasted between 58 and 90 min were audio-recorded and transcribed verbatim. The question that unexpectedly brought forth the theme of anxiety was: “What emotions did you experience when you were becoming less religious?” Participants’ responses to this prompt revealed rich and recurring reflections on anxiety, which became the focus of the thematic analysis presented in Study 1.

Participants were recruited through an announcement on the first author’s Facebook profile. Interviews were conducted online or via mobile applications. Each participant gave informed consent to participate, with additional parental consent obtained for underage participants. Participants received a voucher worth PLN 50 (approximately USD 12.41) as compensation for their time.

#### Researcher Reflexivity

An essential component of reflexive thematic analysis is the explicit consideration of how researchers’ backgrounds, perspectives, and positioning shape the research process (Braun & Clarke, 2021). Rather than seeking to bracket or eliminate subjectivity, we acknowledge it as a valuable epistemological resource that contributes to meaning-making and enriches the analytic process. Our interpretations were informed by our respective disciplinary orientations, life experiences, and ongoing reflexive dialogue throughout the study.

Both authors are psychologists and researchers specializing in the psychology of religion, with a deep familiarity with the Polish cultural context and the Roman Catholic tradition, in which they were both raised. This cultural embeddedness allowed for a contextually sensitive engagement with participants’ narratives.

The first author is a PhD candidate with academic training in both psychology and theology. He also has over 16 years of practical experience in pastoral work with adolescents and young adults. His professional background includes close engagement with youth navigating questions of belief, identity, and meaning. His interdisciplinary education allows for an integrative understanding of religious language and existential themes in participants’ narratives. His ability to build trust and relational depth enriched the interview process, while his reflexive stance contributed to the interpretative quality of the analysis.

The second author is a psychologist and experienced academic specializing in the psychology of religion. For over two decades, she has conducted empirical research on religious transformation, specifically focusing on deconversion and spiritual change. Her expertise is grounded in developmental approaches, with a focus on meaning-making and identity processes. She has published extensively in this field, and her theoretical and analytical insight has contributed to the conceptual clarity and depth of the study.

In line with reflexive methodology, we also considered how our interactions with participants—and the relational dynamics of the interview context—may have influenced the data (Alam & Asmawi, 2024; Levitt et al., 2017). We invited participants to move beyond direct questions and share the meaning-laden aspects of their experiences, allowing for richer narratives to emerge. Throughout the analysis, we engaged in regular collaborative discussions, examining how our respective perspectives shaped what we noticed, prioritized, and interpreted. Our interdisciplinary collaboration, grounded in differing yet complementary professional and academic backgrounds, fostered a dialogical approach that enhanced the depth, credibility, and transparency of our findings.

#### Data Analysis

The data gathered from interviews were examined using qualitative content analysis, as suggested by Graneheim and Lundman (2004). They stated that qualitative content analysis can provide a level of interpretation that reveals themes addressing the question “How” and represents “a thread of underlying meaning” (p. 107). We conducted our content analysis using a conventional method frequently applied in exploratory research within less-studied fields (Green & Thorogood, 2004; Hsieh & Shannon, 2005). This approach relies on the unique insights of participants and the data itself (Hsieh & Shannon, 2005), facilitating transparent reporting of recurring themes that emerge from the data (Green & Thorogood, 2004).

The analysis followed the six-step process (Graneheim & Lundman, 2004). The authors (1) read the interview transcripts several times to understand the content fully. Next, (2) they extracted texts related to experiences of religion, deconversion, and anxiety, combining them into a single text to create a cohesive analysis unit. (3) The text was segmented into meaning units, which were then condensed. (4) The condensed meaning units were generalized, and each unit received a corresponding code. (5) The meaning units, along with the codes, were discussed and reflected on to achieve a consensus on the classification of the codes. (6) Finally, themes were developed that represented the underlying constructs of the categories.

At each stage of the analysis, we employ reliability measures as recommended in the literature (Nowell et al., 2017). To enhance the credibility and robustness of this study and its results, the procedures for conducting interviews, analyzing data, reporting, and interpreting findings were carried out in consultation with independent researchers. These researchers were not involved in any phase of this study investigation. This approach was adopted to introduce an analytical perspective from an external expert (Robinson, 2014).

### Results

Three major themes were constructed through reflexive thematic analysis: Confronting Turmoil at the Beginning, Mourning the Loss of Foundational Beliefs, and Progressing toward Adaptation. Table 2 presents a summary of the three themes, including brief descriptions, illustrative participant quotes, and their frequency of occurrence across interviews. A more detailed account of each theme, along with extended participant quotations, is provided in the online supplemental materials.

**Table 2:** Summary of Themes, Descriptions, Example Quotes, and Frequencies

Theme	Description	Example quotes	Frequency n (%)
Confronting Turmoil at the Beginning	Participants described intense emotional distress during the initial phase of deconversion, often driven by fear of divine punishment and the loss of God’s favor. Letting go of religious beliefs was experienced as losing a source of comfort and safety, which led to feelings of helplessness and isolation. These spiritual fears were compounded by anxiety over potential judgment and rejection from their former religious community.	The idea that leaving the church meant I would go to hell stuck with me and was terrifying. (P1). I was scared. I kept thinking, 'What’s going to happen next?' If I pull away from God, will I be able to handle it? Will I be punished? And honestly, does any of this make sense? (P3). I felt very anxious and upset. I worried about why this happened and what others would think if I left the church. It wasn't easy, especially since I knew so many people in that community and was deeply involved. The thought of leaving them caused me constant stress. (P4)	5 (100%)
Mourning the Loss of Foundational Beliefs	Participants described their deconversion as emotionally ambivalent, marked by both relief and freedom as well as uncertainty and fear. Some expressed curiosity about life within their former faith, while others struggled with anxiety and a loss of meaning once tied to religion	I have had all kinds of thoughts. One day, I felt like I was doing the right thing, like I could finally be free. I was genuinely happy. But then the next day, I had different thoughts, wondering if I was making a huge mistake and if maybe religion and the church were right. That was really annoying, though. (P5 I doubted a lot and often tried to convince myself to return to faith. So I felt lost. Sometimes, I thought it was the right decision because I felt free. But then it was fear. (P1)	4 (80%)
Progressing toward Adaptation	Participants’ narratives underscored the vital role of time in easing the intense emotional struggles tied to deconversion. Initially marked by fear, guilt, and anxiety, these difficult feelings gradually softened as individuals came to terms with their new beliefs and lifestyles.	But over time, those emotions started to fade away. They got weaker. Honestly, I think I even stopped thinking about it. I realized that nothing terrible happened in my life. And that is when I started wondering how much God influences people’s lives. (P4) Once I started doubting, the thought of there being nothing scared me deeply. Over time, though, those fears lessened. I learned to put those worries aside, and they don’t consume me like they used to. (P1)	4 (80%)

The first theme, Confronting Turmoil at the Beginning, highlighted the emotional struggles faced by respondents during the early stages of their deconversion. They expressed feelings of anxiety and stress due to fears of losing God’s favor and potential punishment. Participants reported intense worries about losing social networks, facing judgment, feeling rejected, and managing strained relationships within their religious community. This sense of rejection often extended beyond the community, impacting family relationships. Participants experienced significant anxiety about possibly disappointing their loved ones’ expectations, which led to profound feelings of guilt.

The second theme, Mourning the Loss of Foundational Beliefs, portrayed deconversion as a journey characterized by profound emptiness and mourning for a spiritual foundation. Adolescents expressed sadness over leaving their faith and a longing for the meaning and closeness they once experienced in their relationship with God. They remembered earlier comfort that has now been replaced by painful emptiness. With a nostalgic tone, they reflected on the spiritual support that had provided them with a sense of belonging and inner peace. Their grief over this loss stemmed primarily from imagining how their lives might have unfolded had their faith continued. In these reflections, they yearned for the security and care that had previously felt assured. Thus, this theme signifies a deep loss in deconversion, creating a lingering emptiness where previous meaning once existed.

The third theme, Progressing toward Adaptation, suggested that the intensity of emotions associated with deconversion diminished over time. The feeling of emptiness was no longer as painfully acute. The fear of repercussions from God and rejection by religious individuals lessened. Guilt gradually weakened. Young people became indifferent to religion, slowly redefining themselves and moving toward a new identity centered around unbelief.

### Conclusion

The qualitative findings suggest that adolescent deconversion involves emotional distress, with anxiety most pronounced in the early stages of religious disengagement. This anxiety, linked to fears of divine punishment and social rejection, gradually diminished over time, indicating psychological adaptation to a non-religious identity. These results highlight the emotional dynamics of deconversion and the enduring psychological impact of formerly central religious commitments.

## Study 2. A Longitudinal Analysis of Anxiety and Centrality in Youth Deconversion

Building on the qualitative findings from Study 1, Study 2 was designed to examine the relationship between deconversion and anxiety over time, as well as the moderating role of the centrality of religion in this process. The exploratory design of the qualitative phase allowed us to identify anxiety as a salient emotional response in the process of leaving religion. Participants described anxiety as most intense at the early stages of religious disengagement, gradually diminishing as they adapted to a new belief system. These findings informed two hypotheses:

### Hypothesis 1 (H1)

There is a positive correlation between deconversion and anxiety.

### Hypothesis 2 (H2)

The link between deconversion and anxiety is strongest in the early phases of religious disengagement and gradually diminishes as individuals adjust to their new belief system.

An additional pattern that emerged from Study 1 concerned the prior personal importance of religion in shaping the intensity of emotional responses. Adolescents who had previously viewed religion as central to their identity and worldview reported greater psychological disruption during deconversion. This observation led us to include the centrality of religion as a moderator in Study 2. The centrality of religion refers to the significance of religiosity in terms of personal importance (Huber et al., 2012). For those with a high degree of religious centrality, their faith serves as a fundamental aspect that shapes their identity, values, and purpose in life. Their religious motivations are both stable and deeply ingrained, evident in a self-concept influenced by religion, consistent religious practices, and emotionally significant spiritual experiences. Conversely, individuals with low religious centrality view religion as a marginal aspect of their meaning system. Their interactions with religion are typically situational, influenced by external factors, or driven by non-religious motivations. In these cases, religious behaviors tend to be intermittent, often arising from cultural, familial, or social expectations rather than personal convictions (Zarzycka et al., 2020a, 2020b).

From this perspective, disaffiliation from religion is likely to be more emotionally disruptive for individuals with high centrality, as it involves the loss or reconfiguration of a personally significant and motivationally central framework. The deconversion process for such individuals may challenge core aspects of self-understanding, moral orientation, and meaning-making. Prior research has shown that leaving high-commitment religious groups can be associated with poorer health outcomes, likely due to the loss of tightly integrated social support and shared meaning systems (Scheitle & Adamczyk, 2010). This supports the relevance of examining the centrality of religion as a moderator of the emotional consequences of deconversion. Therefore, we formulated a third hypothesis:

### Hypothesis 3 (H3)

The relationship between deconversion and anxiety is moderated by the centrality of religion, demonstrating a stronger connection for individuals with high centrality compared to those with low centrality.

We implemented a longitudinal design with three data collection points spaced 6 months apart, including an examination of the moderating role of the centrality of religion. Since deconversion is conceptualized as a gradual psychological process involving shifts in identity, worldview, and emotional experience (Paloutzian et al., 2013; Streib et al., 2009), a longitudinal approach was particularly suited to capturing these dynamics over time.

### Method

#### Participants and Procedure

The survey focused on adolescents and was conducted by the first author in secondary schools located in central Poland. In the Polish education system, students can pursue further education in a specific profession or industry through a basic vocational school, or they can attend secondary school after completing primary school. Secondary education can take place in a four-year high school or a five-year technical school, both of which culminate in a matriculation examination (akin to the French baccalauréat). Students from these three types of secondary schools participated in the survey.

Data were collected from September 2022 to September 2023 at three points with 6-month intervals. The choice of six-month intervals over one year was informed by prior longitudinal research on religious doubt and anxiety (Wilt et al., 2017). Additionally, studies on adolescent spirituality used similar time frames to observe spiritual and psychological changes in adolescence (e.g., Kor et al., 2019). This interval provides a balanced approach, sufficient to capture developmental processes in adolescence while also being feasible within the constraints of school-based data collection and the structure of a doctoral research project.

Participants completed a structured questionnaire using a paper-and-pencil approach. All participants were informed of the study's purpose and their option to withdraw at any stage. The respondents provided their informed consent to participate in the study. Researchers also secured consent from the parents of the subjects and the principals of the schools involved in the study.

The total sample at Time 1 consisted of 602 participants who identified as Polish, including 304 females (50.5%) and 288 males (47.8%), with a mean age of 14.51 years (*SD* = 0.79), ranging from 13 to 16 years. All participants were secondary school students. Regarding religious affiliation, 455 identified as Catholic (75.6%), and eleven as Greek Catholic (1.8%). Additionally, 47 participants identified as atheists, 19 as agnostic, and 47 as having no religious affiliation. During the second wave of assessment (T2), 119 participants withdrew from the study, leaving a remaining 484 individuals, which corresponds to an attrition rate of 19.4%. Subsequently, in the third wave of assessment (T3), another 58 participants discontinued their participation, thereby reducing the sample to 426 individuals, resulting in a cumulative attrition rate of 29.35%.

The number of participants in the three assessments varied due to fluctuations in school attendance, instances of withdrawal from the study, and the researcher’s decision to exclude questionnaires that were completed incorrectly. The final sample comprised 268 participants who completed all assessments (*N* = 268). Table S1 in the supplementary materials presents a comparison between the baseline group at T1 and those who completed all assessments. The participants in the three measurements were older than the 602 respondents at T1 (*p* < 0.001). This discrepancy is to be expected, considering the longitudinal design of the study, in which the final assessment occurred one year after the initial phase. No differences were identified in the remaining sociodemographic variables, including gender, place of residence, educational attainment, and religious affiliation. The Study 2 dataset is available in the Open Science Framework (OSF) at https://osf.io/qrjyn/?view_only=7083499da8864a75971fa136fe8ec806.

#### Measures

##### Deconversion

To assess deconversion at T1, T2, and T3, we used the 27-item Adolescent Deconversion Scale (ADS, Nowosielski & Bartczuk, 2017). The ADS consists of five subscales: (1) Abandoning Faith (e.g., *I have begun to doubt that God exists*), (2) Withdrawal from the Community (e.g., *The religious community (Church) is becoming less and less important to me*, (3) Experiencing Transcendental Emptiness (e.g., *I have begun to experience emptiness in my religious life*), (4) Moral Criticism (e.g., *Religious moral principles seem more and more impractical to me*), and (5) Deconversion Behavior (e.g., *I rarely attend religious/spiritual services*). Participants were instructed to consider the last 12 months when responding to the ADS items. The response options ranged from 0 (*completely untrue about me*) to 3 (*very true about me*). We measured the intensity of deconversion processes by using the total score, which is the sum of the subscale scores. Reliability at T1 is satisfactory (*α* = 0.95).

##### Anxiety

We used the 20-item Trait Anxiety subscale of the State-Trait Anxiety Inventory (STAI; Spielberger et al., 1983) to assess the general tendency to feel anxious at T1, T2, and T3 (e.g., *I worry too much over something that really doesn’t matter*). Response options ranged from 1 (*not at all*) to 4 (*very much*). Reliability at T1 is satisfactory (*α* = 0.90).

##### Centrality of Religion

We assessed the centrality of religion at T1, T2, and T3 using the 15-item Centrality of Religion Scale (CRS; Huber & Huber, 2012; Zarzycka et al., 2020a, 2020b). The CRS measures the importance of five aspects of religiosity: intellect, ideology, religious experience, public practice, and private practice. We used the total score from the 15-item CRS, with responses rated on a scale of 1–5 (e.g., *To what extent do you believe that God or something divine exists?*). One question offers eight options, while another provides nine; this variation does not affect the total score. Reliability at T1 is satisfactory (*α* = 0.90).

#### Statistical Analyses

Descriptive statistics and correlations were analyzed using IBM SPSS Statistics 27. A linear mixed model (LMM) was employed in R (version 2024.04.2 + 764) using the lme4 package (Bates et al., 2015) with restricted maximum likelihood (REML) estimation to examine the hypothesized relationships. The dependent variable was anxiety level (Anxiety). Deconversion (Deconversion), time (Time), and the centrality of religion (Centrality) were included as fixed effects, along with their two-way and three-way interactions. Deconversion, Time, and Centrality were mean-centered to enhance interaction interpretation and reduce multicollinearity between interaction terms and main effects (Enders & Tofighi, 2007). Centering these predictors ensures that interaction terms reflect deviations from their respective means rather than arbitrary scale values, facilitating a clearer interpretation of the main effects. A random intercept for participants (ID) was specified to account for intersubject variability. Significance testing was conducted using the lmerTest package (Kuznetsova et al., 2017), which applies the Satterthwaite method to approximate degrees of freedom and calculate p-values for mixed models. The model was specified as follows: Anxiety ~ Deconversion + Time + Centrality + (1|ID).

### Results

At each time point, deconversion had a positive correlation with anxiety measured concurrently. This connection was most robust at T1, diminished at T2, and reached its lowest point at T3. At each time point, the centrality of religion was negatively correlated with deconversion. Furthermore, the centrality of religion at T1 showed a positive correlation with anxiety at T2 (Table 3).

**Table 3:** Descriptives and correlations between the study variables

	Variable	1	2	3	4	5	6	7	8	9
1	Anxiety T1	–								
2	Anxiety T2	0.13*	–							
3	Anxiety T3	− 0.02	−0 .05	–						
4	Deconversion T1	0.31**	0.01	− 0.05	–					
5	Deconversion T2	0.03	0.27**	0.15*	0.08	–				
6	Deconversion T3	− 0.17**	0.04	0.19**	− 0.08	0.05	–			
7	Centrality T1	− 0.01	0.14*	0.01	− 0.61**	0.03	0.03	–		
8	Centrality T2	0.07	− 0.11	− 0.11	0.01	− 0.53**	− 0.06	0.13*	–	
9	Centrality T3	0.12	− 0 .04	− 0.02	0.04	0.02	− .45**	− .04	− 0.01	–
	M	2.37	2.39	2.38	1.14	1.18	1.19	2.63	2.65	2.45
	SD	0.53	0.54	0.52	0.77	0.83	0.85	0.90	0.90	0.88

*p < .05, **p < .01

**Table 4:** Results from the LMM model assessing the effects of deconversion, time, and centrality of religion on anxiety, including interaction effects

Predictor	Estimate	SE	*df*	*t*	*p*	LLCI	ULCI
Intercept	2.44	0.056	738.32	43.286	< 0.001	2.33	2.55
Deconversion	0.383	0.082	787.57	4.699	< 0.001	.22	0.54
Time	− 0.01	0.026	637.32	− 0.394	0.693	−0 .06	0.04
Centrality	0.152	0.072	790.98	2.099	0.036	0.01	0.29
Deconversion × Time	− 0.069	0.037	788.89	− 1.884	0.060	− 0.14	0.00
Deconversion × Centrality	0.165	0.076	792.99	2.18	0.029	0.02	0.31
Time × Centrality	− 0.029	0.032	792.99	− 0.901	0.368	−0 .10	0.03
Deconversion × Time × Centrality	− 0.033	0.034	791.42	− 0.984	0.325	−0 .10	0.03
Variance (Intercept | ID)	0.005						
Residual	0.255						

*SE*  standard error; *LLCI*  lower limit of the 95% confidence interval for the regression coefficient; *ULCI * Upper limit of the 95% confidence interval for the regression coefficient

The LMM results (Table 4) revealed significant main effects of both deconversion (*b* = 0.38, SE = 0.08, *t*(787.57) = 4.70, *p* < .001) and the centrality of religion (*b* = 0.15, SE = 0.07, *t*(790.98) = 2.10, *p* = .036) on anxiety, indicating that higher levels of deconversion and greater religious centrality are associated with increased anxiety.

**Fig. 1:**
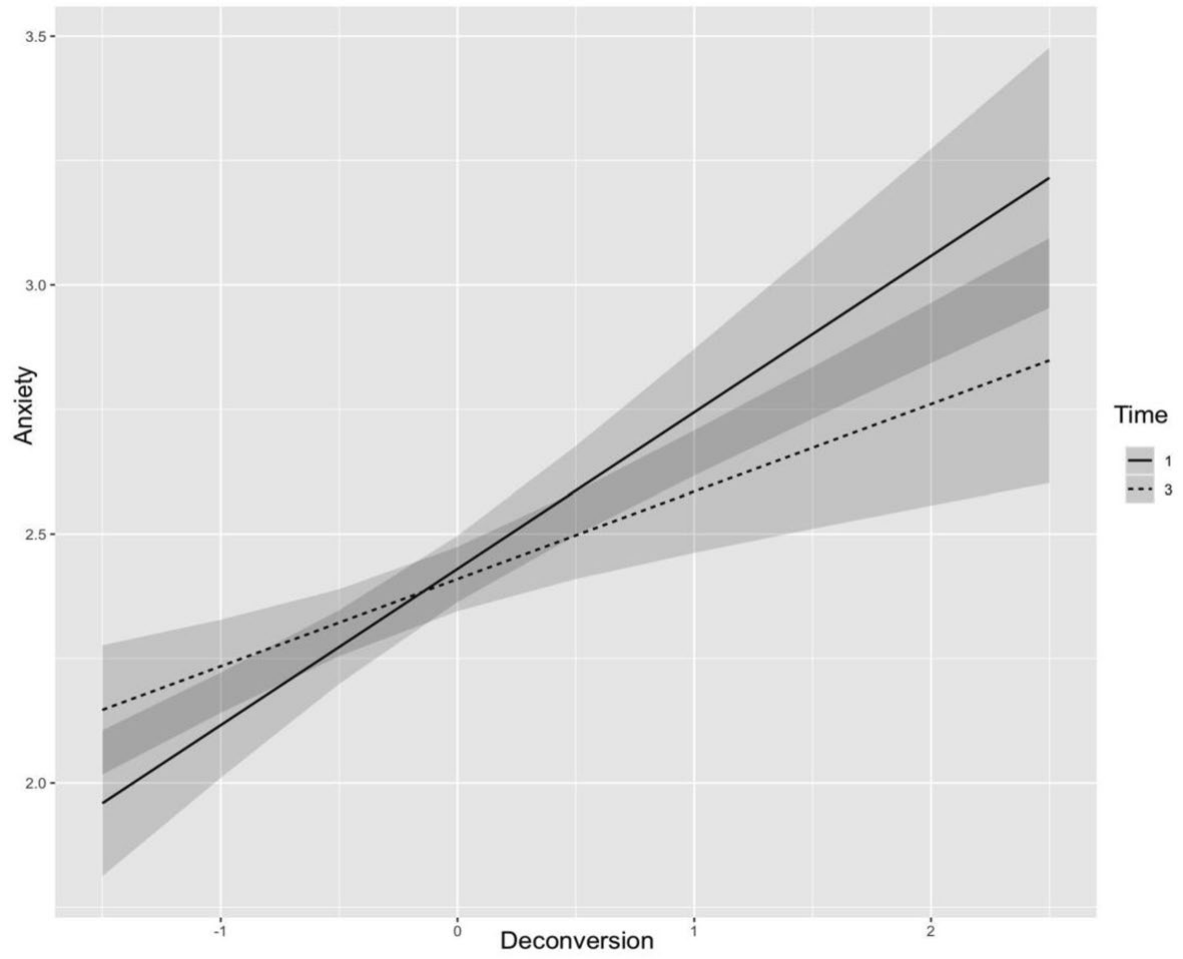
Predicted anxiety levels as a function of deconversion, with time as a moderating factor

The interaction between deconversion and time approached significance (*b* = − 0.07, SE = 0.04, t(788.89) = − 1.88, *p* = .060), suggesting a potential trend toward a weakening relationship between deconversion and anxiety over time. This pattern implies that adolescents may gradually experience reduced anxiety as they move away from their religious beliefs. Figure 1 shows this trajectory.

**Fig. 2:**
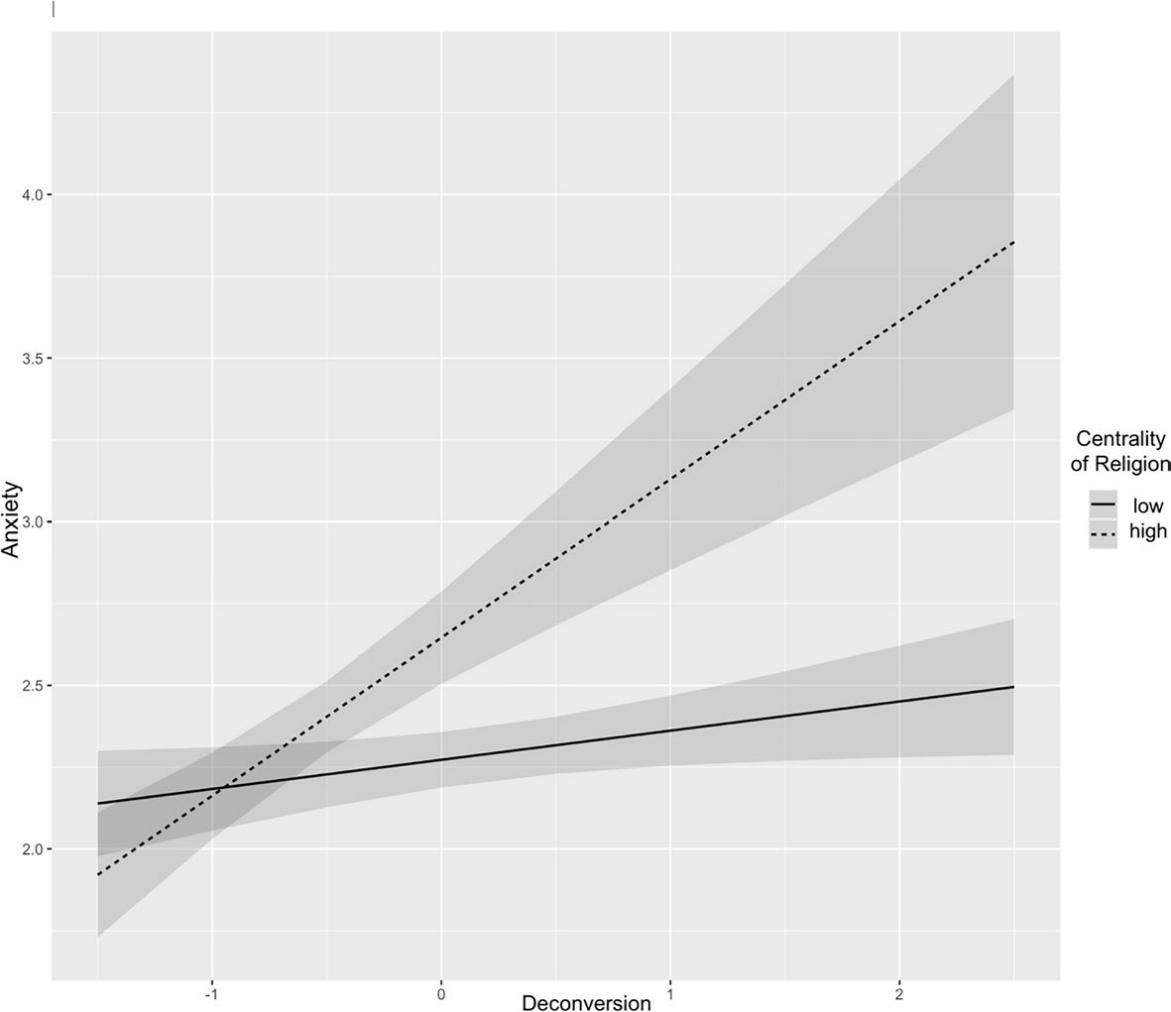
Predicted anxiety levels as a function of deconversion, with the centrality of religion as a moderating factor

The analysis also revealed a significant interaction between deconversion and the centrality of religion (*b* = 0.17, *SE* = 0.08, *t*(792.99) = 2.18, *p* = .029), indicating that the emotional effects of deconversion vary depending on how central religion was to the individual’s life. For those with high religious centrality, deconversion was more strongly associated with elevated anxiety, whereas for individuals with low centrality, this association was not observed. Figure 2 presents this moderating effect.

**Fig. 3:**
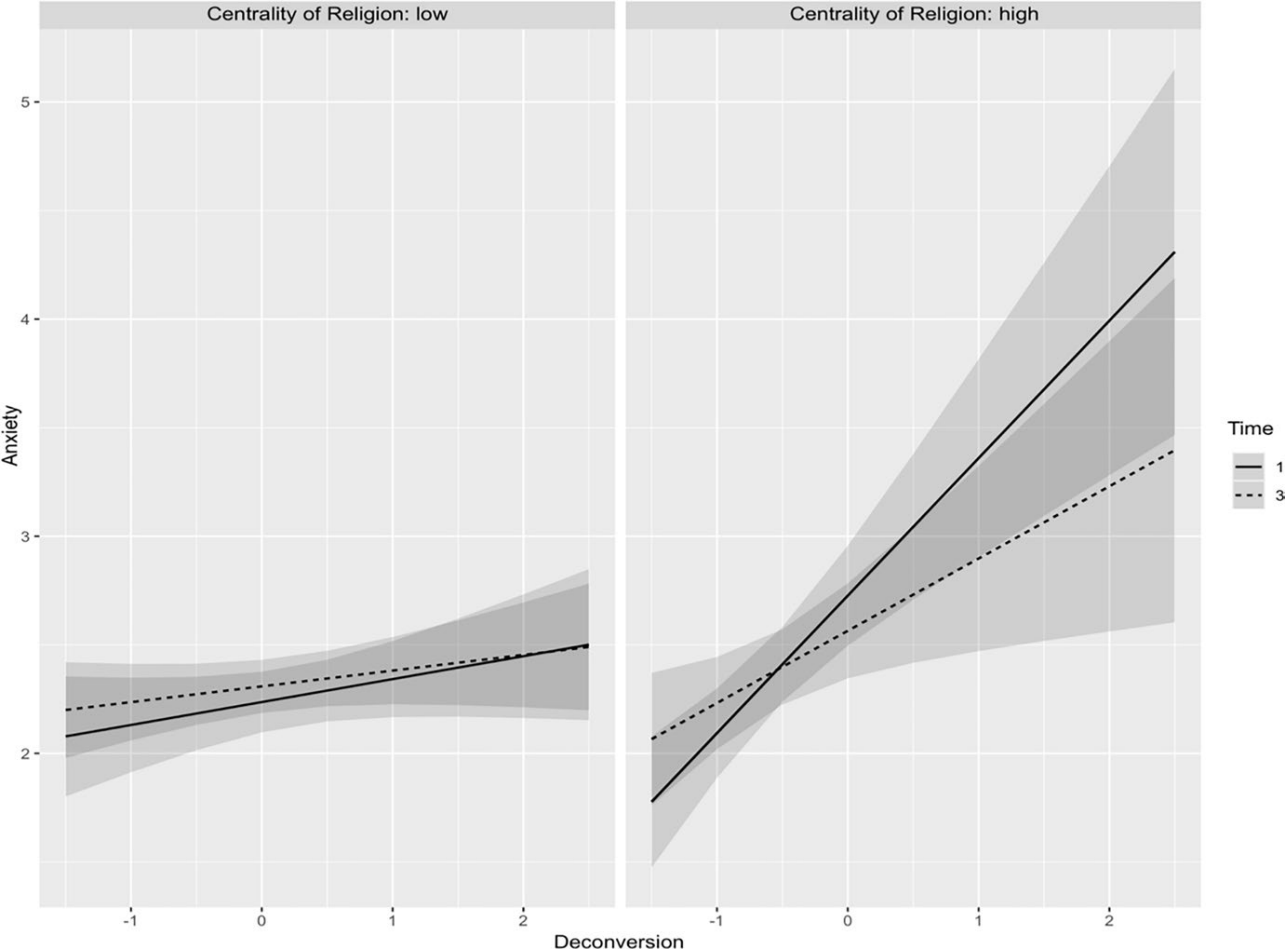
Predicted anxiety levels as a function of deconversion, with time as a moderating factor for individuals with high and low centrality of religion

To further investigate this interaction, a post-hoc analysis was conducted to assess the anticipated anxiety levels among individuals exhibiting low versus high religious centrality. The findings revealed that individuals with low religious centrality had a mean predicted anxiety level of *M* = 2.33, SE = 0.031, 95% CI [2.27, 2.39]. Conversely, individuals with high religious centrality reported a significantly higher anxiety level (*M* = 2.51, SE = 0.038, 95% CI [2.44, 2.59]). The results support the hypothesis that religious centrality acts as a moderating factor, suggesting that the effects of deconversion on anxiety are significantly more pronounced in individuals who view religion as a central aspect. Figure 3 depicts the predicted anxiety levels as a function of deconversion, with time as a moderating factor, presented separately for individuals with high and low centrality of religion.

Model fit diagnostics were performed to evaluate the distribution of residuals, normality, and heteroscedasticity. The model was estimated using restricted maximum likelihood (REML) estimation, resulting in a REML criterion value of 1229.7. The diagnostic results indicated that the residuals were approximately normally distributed, showing only minor deviations, and no significant heteroscedasticity was observed. Additionally, to assess how well the model explains the variance in anxiety levels, marginal and conditional R2 values were calculated. The marginal R2 (R2m), which captures the variance explained by fixed effects, was 0.053, while the conditional R2 (R2c), which includes both fixed and random effects, was 0.055. These values suggest that while the fixed effects contribute to explaining variability in anxiety, additional unmeasured factors likely influence the outcomes. Detailed diagnostic plots and statistical assessments are available in the supplementary materials (S2).

### Conclusion

Deconversion was associated with elevated anxiety, but this association weakened over time. The effect was strongest among adolescents for whom religion was highly central, underscoring the moderating role of the centrality of religion. Despite the modest explained variance, the findings highlight the emotional costs of deconversion, particularly for those deeply rooted in their faith. These findings are consistent with the results of Study 1, where participants described increased anxiety at the initial stages of deconversion, especially when religion had previously held personal and social significance. Over time, this anxiety appeared to subside, reflecting a process of gradual psychological adaptation.

## General Discussion

This study examined the emotional trajectory of deconversion among Polish adolescents, integrating qualitative and longitudinal perspectives. Study 1 identified that anxiety peaks at the onset of deconversion, driven by fears of divine punishment, social rejection, and the loss of a spiritual foundation. Over time, these emotions subside, giving way to a sense of indifference. Study 2 confirmed this trajectory quantitatively, demonstrating a strong initial link between deconversion and anxiety that weakened over time. Furthermore, the centrality of religion moderated this relationship: individuals for whom religion was highly central experienced more pronounced anxiety, while those with low centrality showed no such effect.

Study 1 demonstrated that deconversion is a highly emotional experience, especially in its initial phases when anxiety is most intense. Previous qualitative studies have revealed that individuals often experience distress when questioning or leaving their religious identities (Adam, 2009; Jindra & Lee, 2023; Nica, 2020). Those in the process of deconversion reported feelings of anxiety, regret, guilt, and sadness (Adam, 2009; Nica, 2020). Many expressed anger toward organized religion (Adam, 2009) and frequently felt isolated and unsure of their identities (Jindra & Lee, 2023; Lee & Gubi, 2019). Multiple studies emphasized anxiety in this context (e.g., Adam, 2009; Davidman & Greil, 2007; Lee & Gubi, 2019), particularly peaking during the transition or immediately after leaving religion. Study 1 found that the triggers of anxiety are consistent with previous research, primarily tied to the loss of familial connections and a transformed sense of identity and outlook. Participants noted the challenge of redefining themselves and discovering new meaning in their lives (Davidman & Greil, 2007; Lee & Gubi, 2019).

The findings from Study 1 highlighted anxiety as a key factor in the deconversion process, with participants linking their anxiety to the significance they attributed to religion. These insights informed the design of the subsequent quantitative Study 2. Consequently, Study 2 examined the relationship between deconversion and anxiety over time, incorporating the centrality of religion as a moderating factor. In Poland, where Catholicism remains the dominant religion, it holds substantial cultural value (Żemojtel-Piotrowska et al., 2021). Even individuals who are not formally religious often retain a cultural connection to religion, with the broader religious environment shaping the emotional aspects of deconversion. Exiting a religious community can, therefore, be accompanied by tension and conflict (Jindra & Lee, 2023). Study 2 confirmed Hypothesis 1, showing a positive correlation between deconversion and anxiety at three measurement points. This suggests that young people feel anxiety during the process of deconversion from religion.

Hypothesis 2 was also supported, revealing that the association between deconversion and anxiety is most pronounced in the initial stages of deconversion and decreases over time. This finding aligns with previous studies indicating that deconversion is initially experienced as a form of internal stress and emotional turmoil. However, this internal strain lessens, allowing youth to adapt to life beyond the framework of religious affiliation. Adaptation to disbelief has been noted in other studies. Streib and Keller (2022) demonstrated moderate improvements in sense of purpose and self-acceptance over a decade. Jindra and Lee (2023) highlighted differences in identity perception related to the stage of distancing from the church. Individuals in the early, uncertain phase of deconversion—when they had not yet disclosed their abandonment of religious practices to their families—experienced the most significant struggles with their identity. They also felt the greatest fear of tension within the family environment. In contrast, those who had already shared their deidentification with family members felt less tension and enjoyed a more stable sense of identity. Nica (2020) found that for many individuals, once they begin to let go of or transition away from religious ideology, feelings of guilt, shame, and fear diminish considerably. Thus, the initial cost of deconversion is the most intense (Fazzino, 2014; Streib & Keller, 2020), but the decline in anxiety over time suggests an adaptive process in which individuals gradually integrate their new worldview and identity, mirroring previous longitudinal studies (Streib & Keller, 2020).

Hypothesis 3 was also confirmed, indicating that among individuals with high centrality of religion, the relationship between deconversion and anxiety is significantly stronger than for those with low centrality of religion. This suggests that deconversion for people for whom religion is central may be linked to a profound experience of loss. This loss can relate to worldview and identity, addressing existential questions and providing frameworks for understanding the world (Lee & Gubi, 2019). It may involve losing friendships and family, experiencing social loneliness (Adam, 2009), altering rituals, and losing a sense of ultimate meaning (Davidman & Greil, 2007). Research on ex-fundamentalists has revealed that during the process of leaving their former religion, they faced considerable challenges in forming a new sense of identity outside their previous religious community (Nica, 2020). Their sense of loss and the decline of social relationships and support were also pronounced. The social connections built within religion often faded or weakened after leaving it (Nica, 2019). The anxiety associated with distancing from the religious foundations of identity lingers for an extended period, although it diminishes over time. Despite losing their previous roles, individuals redefined their identities within a new social context. For many, these new roles and positions had a positive impact on their well-being (Nica, 2020).

The results align with cognitive dissonance theory (Festinger, 1957), which suggests that individuals experience psychological discomfort when their deeply held beliefs are challenged. The initial surge in anxiety may arise from efforts to reconcile conflicting worldviews before adopting a coherent alternative. Additionally, the loss of previous meaning structures (Park, 2010) likely contributes to the distress observed in early deconversion stages, as religious frameworks provide existential security and moral guidance. Lastly, the lack of effective coping strategies in the initial phases (Pargament, 2007) may intensify anxiety until individuals establish alternative sources of support and meaning.

The Polish context offers an important sociocultural perspective on deconversion. In a country where Catholicism remains deeply intertwined with national identity and social norms (Mariański, 2021), disengaging from religion has unique social and emotional implications. Young people navigating deconversion may face family tensions, social stigma, and existential uncertainty, which amplify the initial emotional burden. These findings contribute to a broader discourse on religious transformation in contexts where faith is not merely a personal choice but a cultural expectation and where deconversion signifies not just theological doubts but a more extensive renegotiation of identity in a predominantly religious society.

### Limitations

The findings of this study should be interpreted with awareness of several limitations. As with most qualitative research, the findings of Study 1 are not intended to be statistically generalizable (Gheondea-Eladi, 2014). The aim was to gain an in-depth understanding of the emotional experiences of adolescents who have deconverted within a specific cultural context. Although the five selected participants provided rich and thematically saturated narratives and the sampling strategy adhered to the principle of information power (Malterud et al., 2016), the emotional trajectories described may differ in other age groups or in more religiously diverse or secular contexts. Furthermore, the interpretive nature of reflexive thematic analysis means that the positionality of researchers inevitably shapes the construction of themes, despite efforts to maintain reflexive transparency and methodological rigor. Additionally, five participants were recruited through an open call posted on the Facebook profile of the first author. Although this method allowed for voluntary and motivated participation, it also introduces a potential self-selection bias, which limits the representativeness of the sample.

Second, while Study 2 is longitudinal, its duration was relatively short. Future research should investigate emotional adaptation over a longer period, extending beyond one year. Third, the study focused exclusively on anxiety; future research could encompass other responses, such as guilt or relief. Fourth, cross-cultural comparisons provide valuable insights, as the dynamics of deconversion vary among different religious traditions and secular environments. In Poland, disengagement occurs in a culturally uniform environment primarily influenced by Catholicism. The processes of deconversion are shaped by both what individuals abandon and the alternatives present (Streib, 2021). Fifth, in Study 2, we noted a relatively high attrition rate. Many participants did not submit data from all three assessments. The surveys were conducted in classrooms, and much of the missing information resulted from students being absent during one of the three survey phases. A 44.4% attrition rate significantly decreased the sample size throughout the study. This may have affected the effect sizes we obtained.

This study makes significant contributions to the psychology of religion in several important ways. Theoretically, it advances current models of religious disaffiliation by identifying anxiety as a key emotional dynamic in adolescent deconversion and demonstrating how this trajectory is shaped by the prior centrality of religion. Methodologically, the sequential mixed-methods design exemplifies how qualitative insights can inform and structure longitudinal quantitative inquiry, offering a robust model for future research on transitional religious experiences. Practically, the study addresses a socially relevant and underexplored issue in contemporary Polish society. By elucidating the emotional costs of deconversion and the role of religious centrality, our findings provide valuable guidance for mental health professionals, educators, and pastoral workers seeking to support adolescents navigating religious change in culturally Catholic settings.

### General Conclusion

This study highlights the emotional dynamics of deconversion among Polish adolescents, showing that anxiety is most pronounced at the outset of religious disengagement but tends to decline over time. The centrality of religion emerged as a key moderating factor—those for whom religion had been more personally significant experienced greater emotional disruption. Methodologically, the qualitative phase informed the design of the longitudinal study by identifying anxiety as a salient emotional response and suggesting its temporal pattern. This sequential mixed-methods approach allowed for a more nuanced understanding of how emotional adjustment unfolds during deconversion.

## Supplementary Information

Below is the link to the electronic supplementary material.

Supplementary file1 (DOCX 400 KB)

## Data Availability

The materials for this study (anonymized interviews in Polish and selected excerpts in English) can be obtained by emailing the corresponding author.
