# Supplementary material for: The Experience of Deconversion Among Polish Catholic Adolescents: A Mixed-Methods Investigation
Source: J Relig Health. 2025 Aug 29;64(6):4348–71. doi: 10.1007/s10943-025-02428-x (PMC12518483; doi:10.1007/s10943-025-02428-x)

**Supplement to: The experience of deconversion among Polish Catholic adolescents:**

**A mixed-methods investigation**

**S1. The Themes Identified Through Qualitative Content Analysis (QCA) with Direct Anonymous Quotations from the Interviews**

**Theme 1. Confronting Turmoil at the Beginning**

Participants reported feeling emotional turmoil during the initial stages of deconversion. They consistently expressed their fear and anxiety about divine retribution. They were convinced that moving away from their previous beliefs would result in spiritual consequences that plunged them into an emotionally overwhelming state. One participant directly explained the source of his fear, attributing it to the Church's constant focus on hell, which he noted had left a deep and lasting mark on him thoughts.

*What emotions did I feel? Mainly fear—this constant worry about hell. Hell was such an important topic in that church; they talked about it almost all the time. The idea that leaving the church meant I would go to hell stuck with me and was terrifying.* (P1/female)

Departing from faith was linked to losing a perceived source of comfort and support. Participants likened this experience to losing a safety net, which heightened their feelings of helplessness and loneliness. One participant expressed it as follows.

*I felt so alone and scared because God had always been a great source of support and comfort for me. I always believed I could turn to Him during any problem and wouldn’t feel alone. But when I started to pull away, it felt like I lost that support. I couldn’t simply reach out to someone and expect that it might help or that anything could change because of it. I was scared. I kept thinking, 'What’s going to happen next?' If I pull away from God, will I be able to handle it? Will I be punished? And honestly, does any of this make sense?* (P3/female)

Along with fears of spiritual repercussions, young people thinking about leaving the Church often expressed significant worries about those who stayed within their previous religious community. This fear typically arose from anxieties about possible judgment, social rejection, or losing relationships that once held great significance. For many, deconversion represented a transformation in personal beliefs and a breakdown of connections with people who once played a significant role in their social and emotional lives. A participant conveyed considerable psychological distress resulting from such reactions:

*I felt very anxious and upset. I worried about why this happened and what others would think if I left the church. It wasn't easy, especially since I knew so many people in that community and was deeply involved. The thought of leaving them caused me constant stress.* (P4/male).

The sense of rejection extended beyond the immediate community and affected family dynamics. For some, the fear of disappointing family members, especially their parents, weighed heavily on their conscience. One participant described the tension:

*Well, I think it’s mostly how older people view me. They see me as some godless person because I don’t go to church. They wonder how I could do that. There’s also the feeling that I’ve disappointed my parents since they wanted me to believe and attend church with them. But I feel it’s too late to go back now.* (P5/male)

The theme ‘Confronting Turmoil at the Beginning’ highlighted the serious emotional challenges faced by young people leaving religion. Participants frequently reported feelings of anxiety and stress related to fears of losing God’s kindness, withdrawal of blessings, or divine retribution. Alongside concerns about spiritual consequences, participants shared a profound sense of helplessness and loneliness. The process was also marked by intense fears of losing social connections, particularly fears of judgment, rejection, and damaged relationships with members of their former religious community. Both the fear of spiritual consequences and the anticipated reactions from religious community members and family rendered leaving religion a deeply disturbing and emotionally taxing experience.

**Theme 2. Mourning the Loss of Foundational Beliefs**

Teenagers often experience mixed emotions after deciding to leave their religion. They face uncertainty, nostalgia, and a quest for meaning while also enjoying the freedom their choice brings. This emotional ambivalence is sometimes complicated further by anxiety, adding layers to their feelings. One teenager illustrated this struggle:

*I have had all kinds of thoughts. One day, I felt like I was doing the right thing, like I could finally be free. It felt amazing to think I could live by the rules I truly wanted, not the ones that religion imposed on me. I was genuinely happy. But then the next day, I had different thoughts, wondering if I was making a huge mistake and if maybe religion and the church were right. That was really annoying, though.* (P5/male).

This ambivalence was compounded by a curiosity about what life might have been like had they remained within their religious community. At times, participants expressed relief and excitement over their newfound independence. However, this was counterbalanced by moments of fear—fear of making the wrong decision and fear of the unknown. One participant articulated this duality, torn between exploring life beyond religion and imagining the possibilities of staying:

*I was confused. On the one hand, I was curious about what would happen next, wondering what my life would be like. Do I really need to believe? Do I have to practice all the time? Or I could achieve something without all that. But then again, I sometimes think about what life would be like if I, for example, went to church every Sunday, prayed every day… and things like that. I wonder what would change. And I guess there is this feeling of loss. Sometimes, I feel a bit of… I don’t know; maybe there is a sense of missing out. Yeah, I think that is how I would describe it: missing out.* (P2/female).

This emotional conflict often left participants feeling unanchored, questioning whether leaving their faith was the right choice. While some felt liberated from religious obligations, others struggled with the realization that religion had provided many with a sense of purpose and direction that they now felt was missing. This relief often came with moments of anxiety about what lay ahead or the perceived emptiness left by religion. One participant described how this tension led to feelings of self-doubt:

*I doubted a lot and often tried to convince myself to return to faith. So I felt lost. Sometimes, I thought it was the right decision because I felt free. But then it was fear. I would think about how, thanks to religion, many people have a purpose in life, something to believe in, which makes it easier for them to live. And here I am, not believing in anything, so I don’t have a purpose or anything like that. And it made me feel worse about myself.* (P1/female).

The analysis of participants’ experiences reveals that deconversion is a profoundly ambivalent and emotionally charged process marked by significant psychological complexity. Participants navigated a dynamic tension between feelings of liberation and regret, curiosity and longing, hope and fear. These internal conflicts often led them to question their decisions and engage in an intense search for new sources of identity and meaning. The coexistence of relief and anxiety underscores the psychological intricacy of this process, portraying deconversion as a deeply transformative yet inherently destabilizing experience.

**Theme 3. Progressing toward Adaptation**

Participants’ narratives highlighted the crucial role of time in alleviating the negative emotional states linked to deconversion. Initially characterized by fear, guilt, and anxiety, these emotions gradually diminished as individuals adapted to their new beliefs and lifestyles. One participant noted the prevalence of fear during the early stages of deconversion, particularly regarding the potential for negative outcomes and the associated uncertainty.

*I had some negative emotions. Like, I was scared. I thought maybe something wrong would happen because I was not attending church anymore. And I didn’t know what to expect. So, at first, those emotions were... Well, they weren’t easy. I would say they were pretty tough. It wasn’t like I didn’t care at all. I felt like religion still played a role in my life. But over time, those emotions started to fade away. They got weaker. Honestly, I think I even stopped thinking about it. I realized that nothing terrible happened in my life. And that is when I started wondering how much God influences people’s lives.* (P4/male).

Similarly, the guilt and perceived sense of failure associated with leaving religion were initially described as overwhelming but diminished over time, eventually resulting in indifference. One participant reflected:

*At first, I felt guilty, like I failed, like I was such a sinner, and that some punishment was waiting for me. But with time, I stopped caring. It didn’t matter to me anymore. Even now, I think I don’t care. I am not as pessimistic about it as I used to be. There was a time when just hearing the words ‘church’ or ‘religion’ would make me sick. But now not...* (P2 / female)

Existential fears, particularly those related to life after death, played a significant role during the early stages of deconversion but gradually lessened in emotional intensity. This change was linked to adaptation over time:

*At first, when I started to think about it more, I was scared. I thought, ‘Oh my God if you are a Christian, life feels peaceful because you have eternal life after you die.’ But then, when I started questioning it, I had this thought: ‘Oh my God, I’ll die, and there will be nothing after that,’ and it freaked me out. However, over time, I stopped thinking about it. It didn’t bother me as much anymore, and I pushed it aside.* (P3/female).

Time served as a buffer, promoting both cognitive and emotional adjustment. As the initial fears and uncertainties faded, participants felt increasingly ‘less connected’ to the expectations and narratives of their former religious communities. This shift enabled a transition from uncertainty and inner conflict to emotional balance. Gradually, participants began reshaping their identities, liberating themselves from concerns about the perceived consequences of abandoning their faith and ultimately finding peace in their choices. These findings highlight the crucial role of time in navigating the complexities of deconversion, demonstrating how individuals progress from fear and uncertainty to freedom and emotional stability while reexamining their beliefs and nurturing alternative sources of fulfillment meaning.

**Table S1**

*Sample characteristics at Time 1* (*n* = 602) *and attrition effects on participants who completed all three time-points* (*n* = 268)

| Variable | Sample at T1  (*N* = 602) | Final Sample  (*N* = 268) | *p* |
| --- | --- | --- | --- |
| Gender |  |  | .64 |
| Male | 288 (47.8%) | 131 (48.9%) |  |
| Female | 304 (50.5%) | 133 (49.6%) |  |
| Other | 10 (1.7%) | 4 (1.5%) |  |
| Age (M, SD) | 14.51 (0.79) | 14.87 (0.94) | < .001 |
| Residence |  |  | .26 |
| Village | 254 (42.2%) | 130 (48.5%) |  |
| City < 50.000 | 229 (38.0%) | 101 (37.7%) |  |
| City 50.000–200.000 | 106 (17.6%) | 36 (13.4%) |  |
| No answer | 13 (2.2%) | 1 (0.4%) |  |
| Education |  |  | .25 |
| Vocational school | 26 (4.3%) | 16 (6.0%) |  |
| High school | 196 (32.6%) | 104 (38.8%) |  |
| Technical school | 357 (59.3%) | 139 (51.9%) |  |
| No answer | 23 (3.8%) | 9 (3.4%) |  |
| Religion |  |  | .69 |
| Roman Catholic | 455 (75.6%) | 215 (80.5%) |  |
| Greek Catholic | 11 (1.8%) | 6 (2.2%) |  |
| Orthodox | 4 (0.7%) | 0 (0%) |  |
| Protestant | 1 (0.2%) | 0 (0%) |  |
| Old Catholic | 3 (0.5%) | 2 (0.7%) |  |
| Baptist | 1 (0.2%) | 0 (0%) |  |
| Jehovah’s Witness | 2 (0.3%) | 2 (0.7%) |  |
| Agnostic | 19 (3.2%) | 6 (2.2%) |  |
| Atheist | 47 (7.8%) | 19 (7.1%) |  |
| None | 47 (7.8%) | 13 (4.9%) |  |
| Other | 12 (2.0%) | 5 (1.9%) |  |

## S2. Model Fit Diagnostics for Study 2

Diagnostic analyses were conducted to examine the distribution of residuals, normality, and potential heteroscedasticity to assess the validity of the mixed-effects model.

### S2.1. Histogram of Residuals

A histogram of residuals (Figure S3) indicates that the residuals exhibit an approximately normal distribution, suggesting an overall good model fit.

**Figure S1**
*Histogram of residuals from the tested linear mixed model (LMM)*


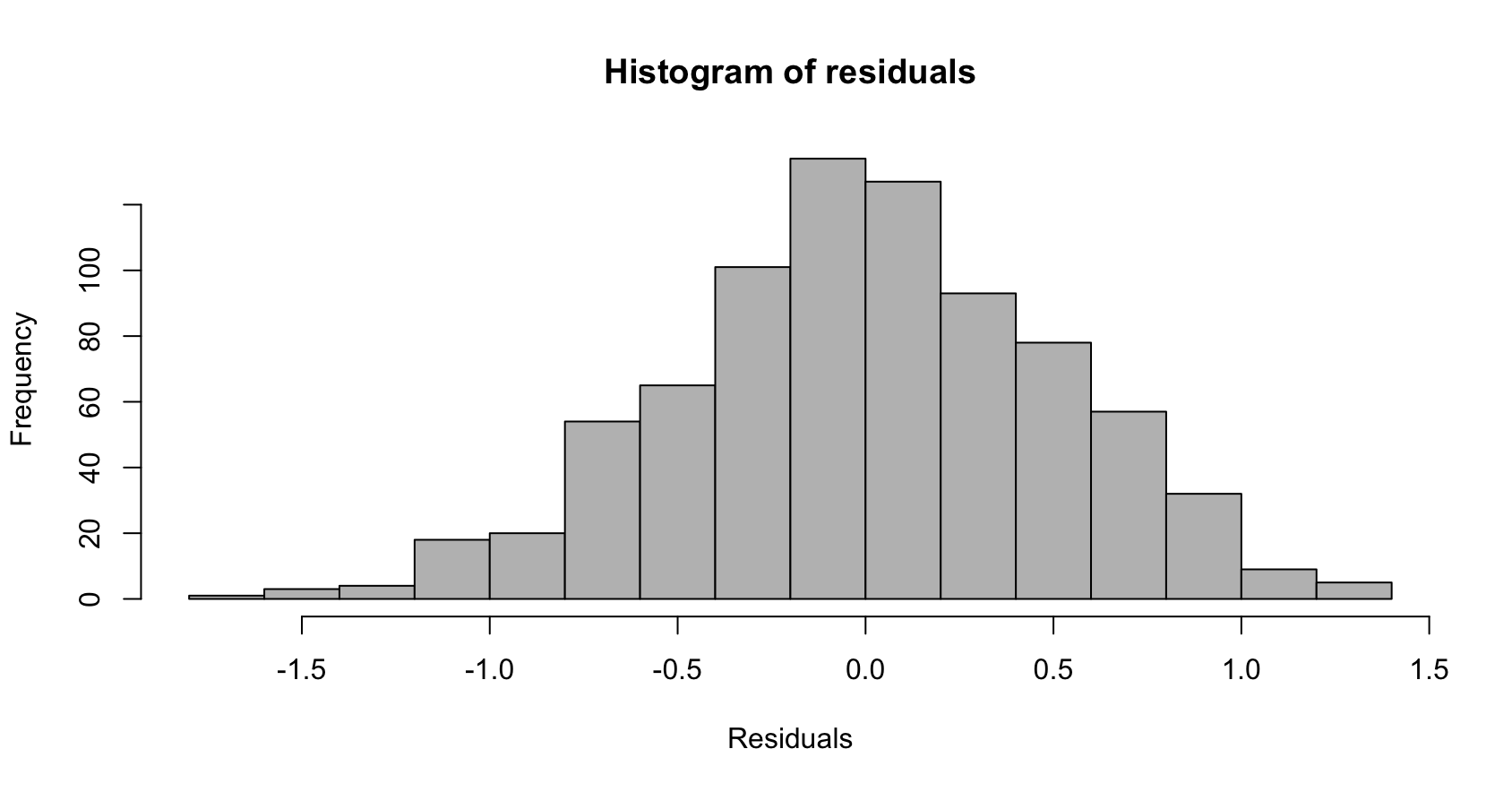


###

### S2.2. Normal Q-Q Plot of Residuals

A Q-Q plot was used to further assess the normality of residuals (Figure S2). The data points closely follow the theoretical normal distribution line, with only minor deviations in the tails, supporting the normality assumption.

**Figure S2**
*Normal Q-Q plot comparing the quantiles of residuals from the tested linear mixed model (LMM) to the quantiles of a theoretical normal distribution.*


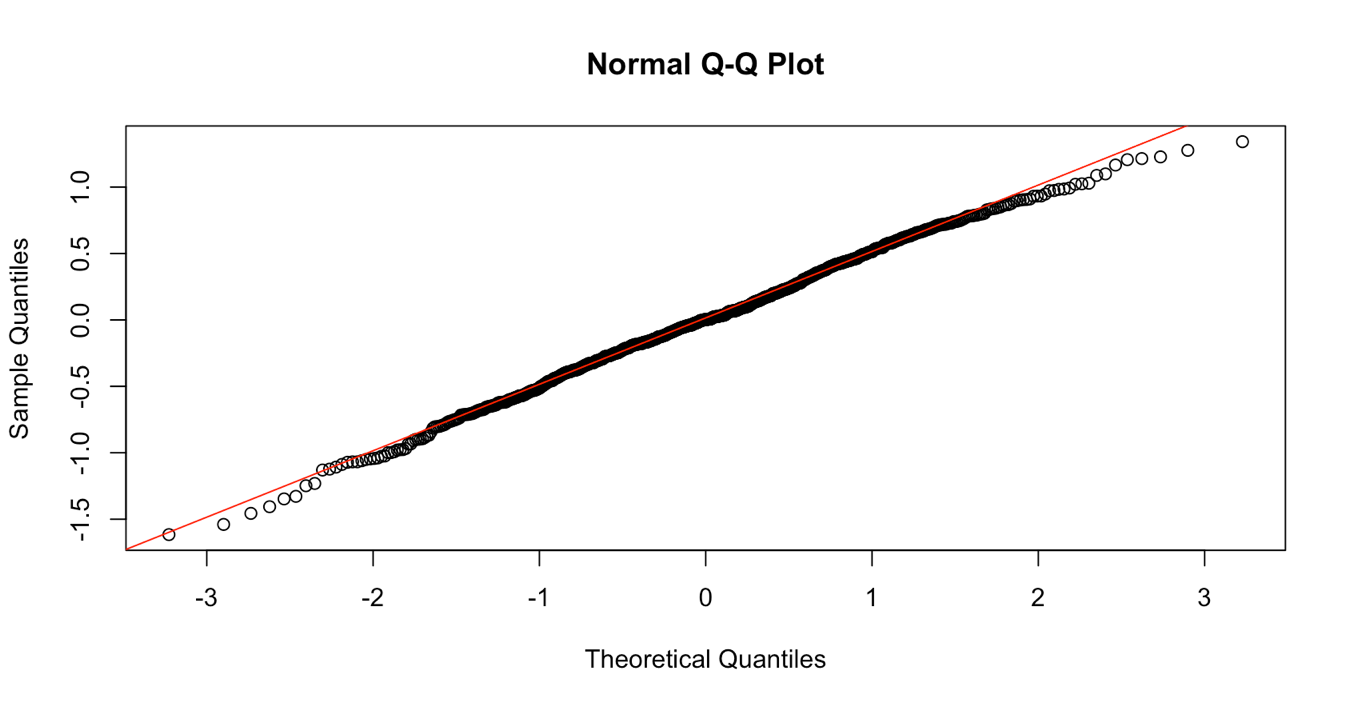


### S2.3. Residuals vs. Fitted Values Plot

The residuals versus fitted values plot (Figure S3) does not indicate any substantial pattern, suggesting that heteroscedasticity is not a significant issue. The residuals appear to be randomly scattered around zero, which supports the assumption of homoscedasticity.

**Figure S3**
*Fitted values vs. residuals for the linear mixed model*

https://link.springer.com/article/10.1007/s10212-024-00802-z
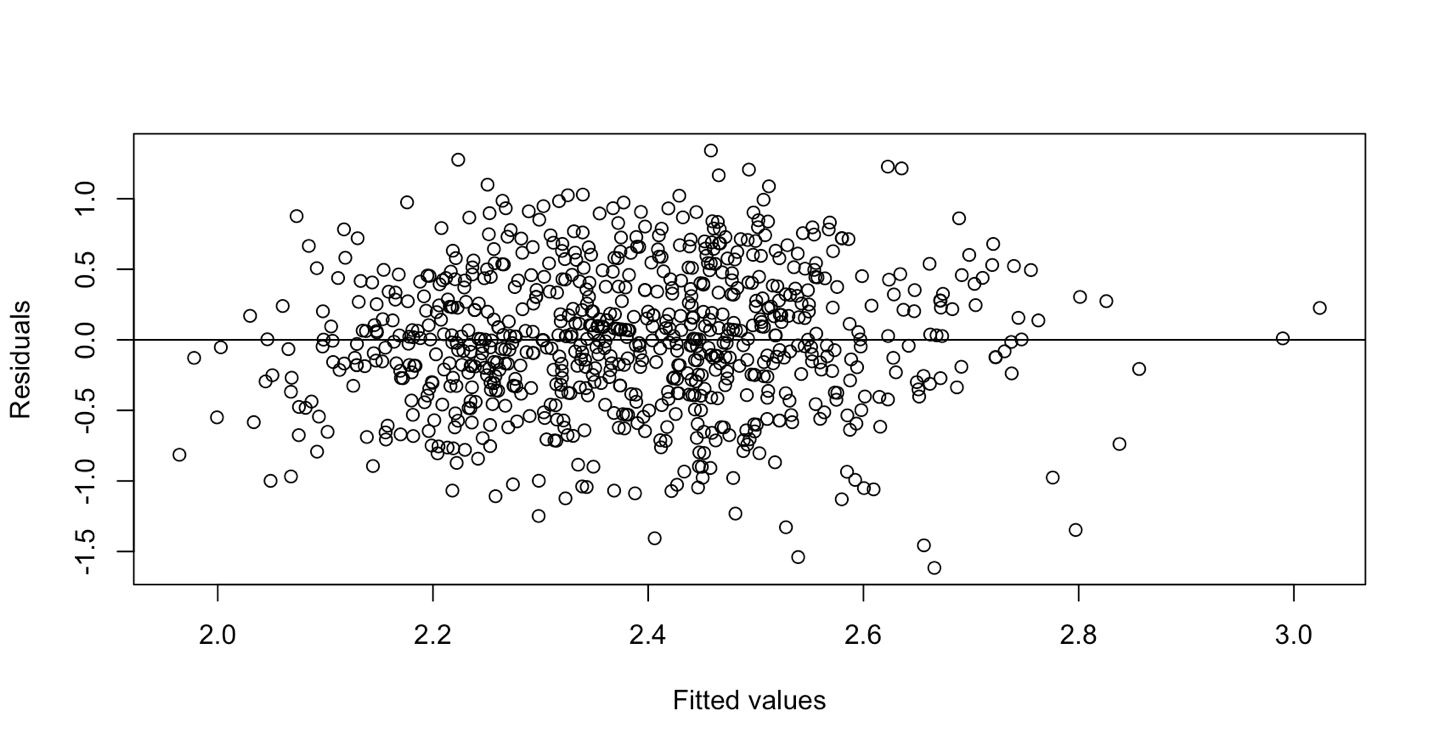

Supplement: Supplementary file 1 — Supplementary file1 (DOCX 400 KB) [file 10943_2025_2428_MOESM1_ESM.docx]
